# Hiat1 as a new transporter involved in ammonia regulation

**DOI:** 10.1038/s41598-023-31503-0

**Published:** 2023-03-17

**Authors:** Sandra Fehsenfeld, Alex R. Quijada-Rodriguez, Haonan Zhouyao, Andrea C. Durant, Andrew Donini, Maria Sachs, Peter Eck, Dirk Weihrauch

**Affiliations:** 1 Département de Biologie, Chimie et Géographie, Université du Quebec à Rimouski, 300 Allée des Ursulines, Rimouski, QC G5L 3A1 Canada; 2 Department of Biological Sciences, University of Manitoba, 50 Sifton Road, Winnipeg, MB R3T 2N2 Canada; 3 Department of Food and Human Nutritional Sciences, University of Manitoba, 35 Chancellor’s Circle, Winnipeg, MB R3T 2N2 Canada; 4 Department of Biology, York University, 4700 Keele St, Toronto, ON M3J 1P3 Canada; 5 Department of Biological Sciences, University of Toronto Scarborough, 1265 Military Trail, Toronto, ON M1C 1A4 Canada

**Keywords:** Physiology, Metabolism, Homeostasis, Zoology, Animal physiology

## Abstract

The orphan transporter hippocampus-abundant transcript 1 (*Hiat1*) was first identified in the mammalian brain. Its specific substrate specificity, however, has not been investigated to date. Here, we identified and analyzed *Hiat1* in a crustacean, the green crab *Carcinus maenas*. Our phylogenetic analysis showed that Hiat1 protein is conserved at a considerable level between mammals and this invertebrate (ca. 78% identical and conserved amino acids). Functional expression of *Carcinus maenas* Hiat1 in *Xenopus laevis* oocytes demonstrated the capability to transport ammonia (likely NH_4_^+^) in a sodium-dependent manner. Furthermore, applying quantitative polymerase chain reaction, our results indicated a physiological role for *Carcinus maenas Hiat1* in ammonia homeostasis, as mRNA abundance increased in posterior gills in response to elevated circulating hemolymph ammonia upon exposure to high environmental ammonia. Its ubiquitous mRNA expression pattern also suggests an essential role in general cellular detoxification of ammonia. Overall, our results introduce a new ubiquitously expressed ammonia transporter, consequently demanding revision of our understanding of ammonia handling in key model systems from mammalian kidneys to crustacean and fish gills.

## Introduction

The orphan epithelial membrane transporter hippocampus-abundant transcript 1 (*Hiat1*) was first described in the hippocampus of rats, where the 490 amino acid, 53 kDa protein was uniformly and abundantly expressed in neonatal rat brain and numerous tissues including testis, lung and kidney^1^. Initially, mammalian *Hiat1,* which contains 12 transmembrane domains, was predicted as a novel sugar transporter due to the presence of sugar transporter-specific motif D-R/K-X-G-R-R/K between transmembrane domain 2 and 3^1^. Only later was *Hiat1* classified as a solute carrier (SLC; facilitative or secondary active membrane transporters independent of ATP) and more specifically as a member of the major facilitator superfamily (MFS)^2^. Meanwhile it has also been shown that at least the mammalian Hiat1 molecules do not promote the transport of glucose^3^.

Recently, Hiat1 (renamed as Mfsd14a in mammals) was found to play an important role in mammalian spermatogenesis^4^. Mutant *Mfsd14a* mice were sterile, and the significantly reduced number of mature sperm produced were round headed as also observed in spermatozoa of humans suffering from globozoospermia^4^. Furthermore, *Mfsd14a* was suggested to play a role in energy homeostasis as its mRNA abundance was significantly down regulated by amino acid starvation^5^. While amino acids are crucial for protein synthesis in all organisms, their catabolism results in the formation of ammonia (herein referred to as the sum of NH_3_ and NH_4_^+^), a critical by-product that exhibits toxic side effects when elevated in the body fluids, e.g., acting as a strong neurotoxin in mammals^6^. Hence, physiological mechanisms must be in place to allow organisms to tightly control systemic ammonia levels. This is for example accomplished by members of another group of facilitative transporters, the SLC42 family (^7^; http://slc.bioparadigms.org/) including ammonia transporters (AMTs), methylammonium permease proteins (MEPs) and Rhesus-glycoproteins (Rh-proteins). As integral membrane proteins AMTs, MEPs and Rh-proteins have been identified to promote ammonia excretion and/or uptake in all phylogenetic groups including bacteria, archaea, fungi, invertebrates, vertebrates and mammals^8–11^. Recently, RNA interference (RNAi)-mediated protein knockdown of AeAMT1 in the sperm of mosquitos (*Aedes aegypti*) has been shown to decrease sperm viability and overall reproduction by being essential for NH_4_^+^-detoxification in germ cells^12^.

Similar to *Hiat1* in mammals, in decapod crustaceans, Rhesus glycoproteins seem to be involved in amino acid metabolism^13^: when a Rh-protein was knocked down in the swimming crab *Portunus trituberculatus*, hemolymph ammonia and amino acids (e.g., glutamine) were significantly increased. Further, in green crabs, *Carcinus maenas*, hypercapnia acclimation is accompanied with decreased Rh-protein mRNA abundance in the gills and elevated ammonia levels^14^ suggesting the downregulation of ammonia transport pathways. Interestingly, a microarray study by Fehsenfeld et al.^15^ identified branchial *Carcinus maenas Hiat1* as one of the most down-regulated genes in response to hypercapnia (GenBank accession no. **DW250260.1**; note that *Carcinus maenas Hiat1* was initially falsely annotated as hyperpolarization-activated nucleotide-gated Na^+^/K^+^-channel (CmHCN2) in the microarray study. CmHCN2 has since been investigated in a different study in green crabs^16^).

Considering (1) AMT’s involvement in ammonia detoxification/transport likely linked to sperm viability, (2) the importance of AMT as a member of facilitative membrane transporters for arthropod male fertility similar to what is seen for Hiat1 in mammals, and (3) the strong downregulation of *Carcinus maenas* Hiat1 in response to hypercapnia when ammonia is known to accumulate and ammonia transporters downregulate, we suggest a role also for *Carcinus maenas Hiat1*/Hiat1 in ammonia detoxification.

Consequently, in the present study, we aimed to characterize the physiological function and substrate specificity of Hiat1 using the green crab *C. maenas*, an established model system to investigate ammonia transport and acid–base regulation^14,17–20^. Based on the above-mentioned findings for hypercapnia exposed green crabs and parallels to Rhesus glycoproteins, we hypothesized that *Carcinus maenas* Hiat1 plays an important role in ammonia regulation and potentially acid–base homeostasis in these crabs by functioning as an ammonia transporter. This study is comprised of a detailed *Carcinus maenas Hiat1* sequence analysis, tissue mRNA profiling, and functional expression of *Carcinus maenas* Hiat1 in frog oocytes to examine NH_4_^+^ transport, applying radiotracer flux experiments and the Scanning Ion-selective Microelectrode Technique (SIET).

## Materials and methods

### Animal care

Male green crabs were caught in the opening of the Pipestem Inlet (Barkley Sound, Vancouver Island, BC, Canada; Department of Fisheries and Oceans collection permit XR-235-2013) and transferred to the University of Manitoba (Winnipeg, MB, Canada). In Winnipeg, animals were kept in artificial seawater (Seachem Marine Salt ®, salinity = 32 ppt.) in recirculating 1200-L tanks at 14 °C and a dark:light cycle of 12 h:12 h, and fed to ad libitum with frozen squid twice a week. Water changes were performed every second day and always on the day after feeding. Green crabs were acclimated to brackish water of a salinity of 10 ppt. for a minimum of 7 days prior to exposure to additional stressors (high *p*CO_2_, high environmental ammonia (HEA)). For acclimation to 10 ppt., 8 crabs each were transferred to smaller 20-L tanks at pH = 7.7 ± 0.0 and *p*CO_2_ = 53.5 ± 2.1 Pa. Subsequently, brackish water acclimated *C. maenas* were fasted for 2 days prior to additional exposure to high *p*CO_2_ and HEA, as well as during the exposure to these stressors. For acclimation to high *p*CO_2_, the IKS Aquastar® was set to control water pH to 7.0 (*p*CO_2_ = 324.3 ± 20.0 Pa) by addition of CO_2_ gas. For acclimation to HEA, the water in the respective tank was enriched with 1 mmol L^−1^ NH_4_Cl and changed daily.

### Sequence-based phylogenetic and structural analysis

To obtain the full sequence for *CmHiat1*, primers were designed based on the expressed sequence tag EST (GenBank accession number **DW250260.1**). Using the RLM-RACE system (First Choice RLM-RACE kit, Ambion, Austin, TX, USA) and gene-specific as well as adaptor primers provided by the kit, the full open reading frame (ORF) was obtained following the manufacturer’s instructions. The gene specific primer for the 5′ end RACE was 5′-TCCCAAGTAAGCTCCCAGTG. The primer 5′-ACAGAGTTGCAGGCTCGATT was used to obtain the 3′ end. The complete *CmHiat1* (GenBank accession no. **MT371392.1**) was then subcloned into the p426 expression vector for amplification in yeast applying sticky-end ligation with T4 ligase (New England Biolabs, Ipswich, Massachusetts, USA) using the restriction enzymes SpeI and SmaI (forward primer including **T7** sequence/SpeI: **TAATACGACTCACTATAGGGactagt**GTGCCTCTGCTGTGGTCAAG; reverse primer containing SmaI: AATcccgggAGCTTTGCATGTGCCTCTTT). Subsequently, the ORF was sent for sequencing at the DNA Sequencing Facility of the Robarts Research Institute (London, Ontario, Canada) using T7/SP6 primers.

For functional expression in *Xenopus laevis* oocytes, the complete *CmHiat1* ORF was amplified from *C. maenas* gill cDNA with Phusion high fidelity DNA polymerase (New England Biolabs, Ipswich, Massachusetts, USA) with primers containing a restriction site for SmaI and EcoRI (forward primer including **T7**/SmaI-restriction site/*ATG*: **TAATACGACTCACTATAGGGcccgggATG**AAGAACAGAGTTGCAGG; reverse primer containing EcoRI: AATgaattcCGCAAACCTGTTCATCAGAA). The ORF was then subcloned into the pGEM®-HE plasmid, a modified pGEM®-3Z vector for the cloning site to be flanked by *Xenopus* beta globin 5′- and 3′- untranslated regions, using T4 ligase as described above. The insertion of the ORF was verified by sequencing with T7/SP6 primers.

For the phylogenetic analysis, the 40 included protein sequences for Hiat1 (Supp. Table 1) were aligned with the MUSCLE algorithm^21^ as provided by MEGA X (default settings^22^). Subsequently, phylogeny was reconstructed applying the method of maximum likelihood based on the Le Gascuel 2008 model and the heuristic method of Nearest-Neighbor-Interchange (NNI) as provided by MEGA X. 1000 replicates were calculated for bootstrapping.

The obtained ORF for *CmHiat1* was used to generate a transmembrane model with Protter^23^ using the default settings. Subsequently, Protter was used to visualise proteoforms for potential phosphorylation sites.

### Quantitative real-time PCR (qPCR)

Total RNA from crab gills and *X. laevis* oocytes was isolated under RNase-free conditions using TRIZOL (Invitrogen, Carlsbad, CA, USA). Following DN*ase* I (Ambion) treatment, DNA-free RNA was transcribed with the iScript cDNA synthesis kit (Biorad, Mississauga, ON, Canada). qPCR was performed on 20 ng cDNA and a final primer concentration of 0.4 μmol L^−1^ in 15 μL reactions with the SSO FastEvaGreen Supermix (Biorad). *Rbs-3* was used for internal normalization of mRNA from hypercapnia-acclimated *C. maenas* in accordance with a recent study in crabs^14^, while elongation factor 1α (*EF1α*) was used for mRNA normalization for HEA-acclimated animals. Additionally, a standard curve was included in each run based on a dilution series of known quantities of the respective gel-extracted gene fragment (0.1 pg–1 fg; QIAquick Gel Extraction Kit, Qiagen). We would like to note that while current MIQE guidelines prefer the application of at least two reference genes, this analysis had been performed before this practice was commonly conducted. More recent mRNA abundance studies by our lab, however, verified *Rbs3* and *EF1α* alone to exhibit a comparable ranking to the combination of up to three reference genes as identified by RefFinder (http://blooge.cn/RefFinder/?type=reference).

The primers for *CmHiat1* (forward: 5′-TGTTTGCTGTCACCTTCAGC; reverse: 5′- TCCCAAGTAAGCTCCCAGTG) were ensured to result in a single specific signal (143 bp) by melting curve analysis following the regular qPCR.

Regular PCR was also performed on *X. laevis* oocytes using gene-specific primers as listed in Supp. Table 2. Ethidium bromide gel analysis (1.5% agarose in 1× TAE buffer) was performed in Apogee Horizon H58 gel chambers to visualize the presence of relevant ammonia transporters *NHE3*, *Rhbg*, *Rhcg* and *XlHiat1*, as well as actin as positive control gene. Gel pictures were taken with the Bio-Rad VersaDoc 4000MP and the Bio-Rad Image Lab™ 3.0 software (Bio-Rad, California, USA) and default settings. *Rhag* was run together with BenchTop 100 bp DNA Ladder (Promega, Madison, Canada) on one gel (panel 1 of Supp. Fig. 4) and zoomed in while acquiring the original image. All other PCR products were run on a second gel where samples for oocyte vs. kidney were divided by the marker. The second image was then separated into panel 2 and 3 of Supp. Fig. 4 and contrast settings of each panel were adjusted in PowerPoint (Microsoft office 365, Washington, USA) to increase visibility of the bands.

### Functional expression of *Carcinus maenas* Hiat1 in *Xenopus laevis* oocytes

#### Chemicals and solutions

[^3^H] 2-deoxyglucose (25.5 Ci/mmol in water) was purchased from PerkinElmer (Waltham, MA, USA). [^3^H]-methylamine hydrochloride (3.1 Ci/mmol, 1 mCi in 1 mL ethanol solution) was obtained from Moravek Inc (Brea, CA, USA). All other chemicals were purchased from Sigma-Aldrich (St. Louis, MI, USA) unless otherwise noted. The standard oocyte ringer (OR2) contained (in mmol L^−1^) 82.5 NaCl, 2.5 KCl, 1 CaCl_2_, 1 MgCl_2_, 1 Na_2_HPO_4_, 5 HEPES, pH 7.5 and was sterilized by vacuum bottle-top filters (EMD MilliporeTM SteritopTM sterile vacuum bottle-top filters, ThermoFisher, Waltham, Massachusetts, USA). Sterile OR2 was supplemented with 2.5 mmol L^−1^ sodium pyruvate, 1 mg mL^−1^ penicillin–streptomycin (Gibco, Long Island, NY, USA) and 50 μg mL^−1^ gentamicin for long term storage of isolated oocytes.

#### Plasmid preparation

The pGEM®-HE plasmid containing the ORF for *CmHiat1*, was transferred into DH5-alpha cells for amplification, column purified (Plasmid miniprep kit, Qiagen, Hilden, Germany) and linearized with SphI (New England Biolabs (NEB), Ipswich, Massachusetts, United States). The HiScribe™ T7 ARCA mRNA Kit (with tailing) (NEB) was used for the in-vitro transcription of the capped mRNA (cRNA) following the manufacturer’s suggestion, column purified (RNeasy MinElute Cleanup Kit, Qiagen, Hilden, Germany) and eluted in nuclease free water.

#### Oocytes preparation

Stage VI-V oocytes were collected from one mature female *Xenopus laevis* (VWR International, Randor, PA, USA) per experimental set-up as previously described^24^. All oocyte expression experiments were repeated on a second batch of oocytes from a different female on the consecutive day. Briefly, the frog was euthanized via decapitation prior to the collection of the ovary. The ovary was placed in Ca^2+^-free OR2 solution containing collagenase type VI (1 mg mL^−1^) (Gibco, Waltham, Massachusetts, USA) while gently agitated for 90 min at room temperature. The activity of collagenase was terminated by rinsing the oocytes three times with standard OR2. Oocytes were then sorted manually, rinsed three additional times with standard OR2 and allowed to recover in sterile OR2 overnight at 16 °C. All procedures used were approved by the University of Manitoba Animal Research Ethics Board and are in accordance with the Guidelines of the Canadian Council on Animal Care, as well as ARRIVE guidelines (https://arriveguidelines.org).

#### Microinjections of oocytes

After the overnight recovery, isolated oocytes were injected with 18.4 ng (36.8 nL with 0.5 ng nL^−1^) of *CmHiat1* cRNA, Human Glucose Transporter 3 (*HsGLUT3*) cRNA as positive control in glucose uptake study, or nuclease-free water as negative control (sham) using the Nanoject II and/or Nanoject III auto-nanoliter injector (Drummond Scientific, Broomall, Pennsylvania, USA). All oocyte experiments were conducted at room temperature two days post-injection.

For the [^3^H] 2-deoxyglucose study (Supp. Fig. 2), oocytes were randomly divided into groups containing twenty oocytes each either injected with water (sham-injected), *CmHiat1* cRNA (CmHiat-expressing), or *HsGLUT3* cRNA (HsGLUT3-expressing). Experiments were performed by incubating oocytes in 200 μL standard OR2 with 125 pmol [^3^H] 2-deoxyglucose for 30 min after which they the experiment was terminated. To terminate the experiment, excess ice-cold standard OR2 was added, followed by four washes with the same solution. Subsequently, oocytes were lysed individually in 200 μL of 10% sodium dodecyl sulfate (SDS) after which 5 mL Ultima Gold scintillation cocktail (PerkinElmer) was added. Internal radioactivity was quantified by liquid scintillation spectrometry (Tri-Carb 2900 TR; PerkinElmer) as counts per min (CPM)/oocyte.

For [^3^H]-methylamine (MA) uptake studies (Fig. 3A), oocytes were equilibrated in standard OR2 and randomly divided into groups containing 15–20 oocytes injected with either *CmHiat1* cRNA (CmHiat1-expressing) or water (sham-injected). Experiments were performed for 60 min in 200 μL standard OR2 adjusted to pH 7.5 and containing 1 mmol (10 μCi) total MA/MA^+^ and terminated as described above. For NH_4_Cl competitive uptake experiments (Fig. 3B), 1 mmol L^−1^ NH_4_Cl was added to the incubation solution. For the Na^+^-free experiment (Fig. 3B), 82.5 mmol L^−1^ NaCl and 1 mmol L^−1^ Na_2_HPO_4_ were substituted by 84.5 mmol L^−1^ choline chloride.

For the [^3^H]-methylamine release study (Supp. Fig. 5), two groups of either twenty CmHiat1-expressing oocytes or sham-injected oocytes were assessed for 0 min and 60 min, respectively. After termination of MA uptake as described above, one group of sham-injected and CmHiat1-expressing oocytes were immediately solubilized to assess the uptake of MA as described above to determine the starting point for the following release experiment (0 h). The second groups of sham-injected and CmHiat1-expressing oocytes were placed in 200 μL of room temperature, radioactivity-free standard OR2 for additional 60 min. After this period, oocytes were washed four times with ice-cold standard OR2, solubilized individually in 200 μL of 10% SDS and assessed for remaining internal radioactivity, quantified by liquid scintillation spectrometry (Tri-Carb 2900 TR; PerkinElmer) as described above.

In all experiments, the efficiency of the washing steps was determined by assessing 50 μL of the radioactivity of the fourth washing post radioactive exposure compared to fresh, radioactivity-free standard OR2 at the same volume.

### Oocyte fluxes as measured by scanning ion-selective microelectrode technique (SIET)

NH_4_^+^, H^+^ and K^+^ microelectrodes for SIET measurements were constructed as described recently^25^. The microelectrodes were calibrated in modified OR2 buffer (composition in mmol L^−1^: ChCl 85; MgCl_2_ 1; Na_2_HPO_4_ 1; CaCl_2_ 1; HEPES 5, pH 7.2) with either 0.1, 1 and 10 mmol L^−1^ NH_4_Cl for NH_4_^+^ measurements, pH 6, 7, and 8 for H^+^ measurements, or 2.5 and 25 mmol L^−1^ KCl for K^+^ measurements. For each flux measurement, an individual oocyte was placed in a well cut out of resin (Sylgard®, Dow Corning, Mississauga, ON, Canada) at the bottom of a 35 mm cell culture dish. The SIET microelectrode was positioned adjacent the oocyte near its circumference where voltage gradients for each ion were measured over an excursion distance of 100 µm. The sampling protocol utilized wait and sample periods of 4 and 1 s, respectively, with the protocol repeated 4 times. Background (noise) measurements were recorded 3000 µm from the oocyte and subtracted from the gradients that were recorded adjacent the oocyte. Resulting gradients were used to calculate fluxes as explained by Chasiotis et al.^8^.

For the NH_4_^+^ and H^+^ kinetics, OR2 buffer contained either 0.5, 5, 10 and 20 mmol L^−1^ NH_4_Cl. Measurements for K^+^ fluxes were performed in modified OR2 buffer with 2.5 mmol L^−1^ KCl and accordingly only 82.5 mmol L^−1^ choline chloride (ChCl) and 0.1 mmol L^−1^ NH_4_Cl, a NH_4_^+^ concentration in which endogenous NH_4_^+^ uptake pathways in *Xenopus* oocytes are inactive^26^.

To verify internal ammonia levels after pre-loading, oocytes were exposed to 20 mmol L^−1^ NH_4_Cl for 60 min and then immediately analyzed for NH_3_/NH_4_^+^, using a gas-sensitive NH_3_ electrode (Orion 9512 from Thermo Scientific, Cambridgeshire, England) connected to a digital mV/pH meter (Supp. Fig. 6A). A second batch of pre-loaded oocytes was used to simultaneously measure and calculate NH_3_/NH_4_^+^ release and H^+^ uptake rates (Supp. Fig. 6B). All SIET oocyte expression experiments were performed on two different batches of oocytes from a different female on different days.

### Statistical analysis

Statistical analyses were performed with GraphPad/Prism 8.1.0 GraphPad Software (San Diego, California USA) or PAST3^27^. Data sets were tested for normal distribution (Shapiro–Wilk test) and homogeneity of variances (F-test) in order to qualify for parametrical testing. If one or both requirements were not met, data was log transformed. Consequently, for comparison of single means, Student’s T-test was applied in Fig. 3A, B and Supp. Figs. 2B, 3 and 6B, and Mann–Whitney-Test was applied in Supp. Figs. 5 and 6A. For the comparison of multiple means, ANOVA with Tukey’s post hoc analysis was used in Fig. 2A, B while Kruskal–Wallis Test with Mann–Whitney pairwise comparison and Bonferroni correction was used in Supp. Fig. 2A. SIET kinetics data (Fig. 4A, B) was analysed by two-way ANOVA with Bonferroni post-hoc multiple comparisons.

All results with P < 0.05 were considered significant. Values are represented as means ± standard error (SE). Graphs were generated using the software GraphPad/Prism 8 and Inkscape, version 0.48 (https://inkscape.org/).

## Results

### Characterization of the *Carcinus maenasHiat1* gene and protein

**Figure 1:**
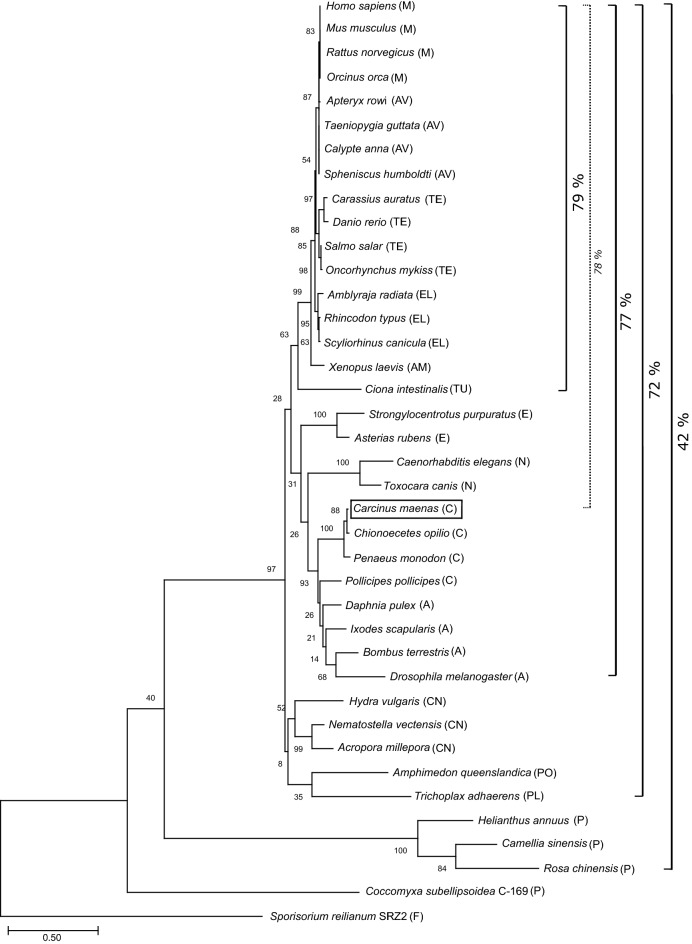
Phylogenetic analysis of *Carcinus maenas Hiat1*. Shown is the Maximum likelihood consensus tree of the MUSCLE alignment of Hiat1 protein as performed with MEGAX^22^. Numbers beside branches represent bootstrap values (1000 replicates). Percentages indicate the level of conservation between respective species (identical + strong residues, as determined by NCBI alignment). The tree is drawn to scale, with branch lengths measured in the number of substitutions per site. A, Arthropoda; AM, Amphibia; AV, Aves; C, Crustacea; CN, Cnidaria; E, Echinodermata; EL, Elasmobranchii; F, Fungi; M, Mammalia; N, Nematoda; P, Plantae; PL, Placozoa; PO, Porifera; TE, Teleostei, TU, Tunicata.

The open reading frame of *Hiat1* in *Carcinus maenas* (*CmHiat1*) translates into a 492 amino acid protein (GenBank accession no. MT371392.1). While CmHiat1 protein forms a distinct clade with other invertebrates including crustaceans, insects, nematodes and echinoderms (Fig. 1), the vertebrate clade with mammals, birds, teleosts, elasmobranchs, amphibia and *Ciona intestinalis* as the most primitive vertebrate at its base, can clearly be distinguished despite Hiat1’s amino acid sequence being conserved at a considerable level between invertebrates and vertebrates. Between *C. maenas* and *H. sapiens*, 306 amino acids (63%) are identical, 77 residues are conserved as strong groups (15%) and 34 residues are conserved as weak groups (7%) so that bootstrap values remain fairly low for this branching (28%). Astonishingly, even compared to the most distant plant *Rosa chinensis*, *C. maenas* exhibits 23% amino acid identity and 29% conservation of strong groups, marking Hiat1 as a conserved protein not only within the animal kingdom but also among eukaryotic organisms in general.

The structure of CmHiat1 as predicted by Protter^23^ shows that the protein possesses 12 transmembrane domains and multiple potential amino acid (aa) phosphorylation sites for protein kinases (PKC = aa12, aa235, aa303, aa326, aa356, aa403, aa452; PKA = aa179, aa214), cyclin-dependent kinases (CDK2/CDK1 = aa2, aa89, aa126, aa34, aa44; CDK5 = aa422) and creatine kinases (CKI = 217, CKII = 144). Furthermore, a phosphorylation site for p38 mitogen-activated protein kinase (p38MAPK) is predicted at position 49, as well as a site for Glycogen synthase kinase 3 (GSK3) at position 167 (Supp. Fig. 1). Contrastingly to initial predictions in the literature, we determined that CmHiat1 is not a glucose transporter, as it was not able to transport ^3^H-Deoxyglucose when expressed in *Xenopus laevis* oocytes (Kruskal Wallis Test with Mann–Whitney pairwise comparisons, P < 0.05, N_sham_ = 19, N_CmHiat1_ = 18, N_HsGLUT3_ = 18; Supp. Fig. 2A).

**Figure 2:**
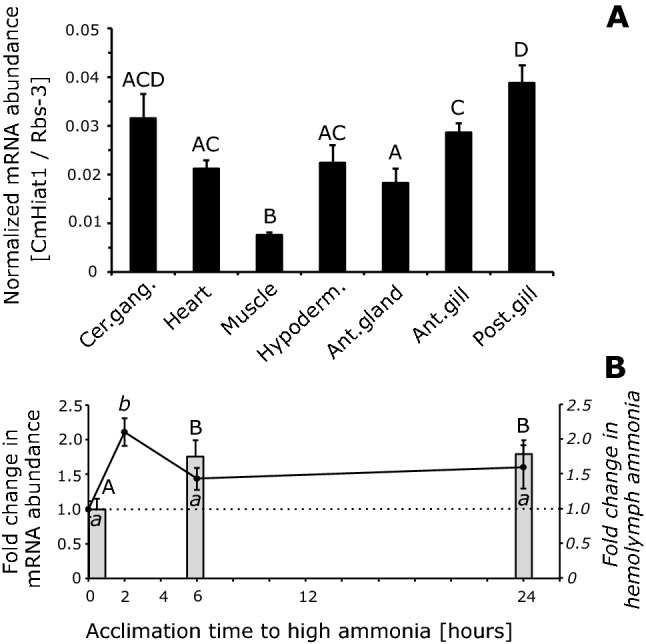
Analysis of *Carcinus maenas Hiat1* mRNA abundance by quantitative PCR. (**A**) Organ and tissue mRNA abundance profile of *Carcinus maenas Hiat1* (black bars); (**B**) Short-term (24 h) acclimation of green crabs to high environmental ammonia of 1 mmol L^−1^ (HEA). Grey bars referred to on the left y-axis in panel (**B**) represent mRNA abundance of *Carcinus maenas Hiat1* in posterior gill #7 at 0, 6, and 24 h of HEA acclimation. Line graph referred to on the right y-axis in panel (**B**) represents fold change in hemolymph ammonia levels after 0, 2, 6, and 24 h of HEA acclimation. *Carcinus maenas Hiat1* has been normalized to either Ribosomal gene 3 (*Rbs-3*, panel (**A**)) or elongation factor 1α (*Ef1*α, panel (**B**)). Upper case letters denote significant differences in mRNA abundance, lower case italic letters denote significant changes in hemolymph ammonia (ANOVA with Tukey’s post-hoc analysis, P > 0.05, N_tissue_ = 5; N_HemoAmm_ = 6; N_RNA,0 h_ = 6, N _RNA,6 h_ = 7, N _RNA,24 h_ = 8). Values are presented as means ± SE. Cer.gang., cerebral ganglion; Hypoderm., hypodermis; Ant. gland, antennal gland; Ant. gill, anterior respiratory gill #5; Post. gill, posterior osmoregulatory active gill #7.

*CmHiat1* mRNA was detected ubiquitously in *C. maenas* across all tissues investigated, with the highest levels of abundance in osmoregulatory active posterior gills and the lowest in muscle (25% expression in muscle *vs*. posterior gills; Kruskal–Wallis with Mann–Whitney pairwise comparison, P < 0.05, N = 5; Fig. 2A). Furthermore, *CmHiat1* mRNA abundance was significantly upregulated in response to short-term exposure to high environmental ammonia of 6 and 24 h (ca. two-fold) (1 mmol L^−1^ for 24 h; ANOVA with Tukey’s pairwise comparisons, P < 0.05, N_0h_ = 6, N_6h_ = 7, N_24h_ = 8), while hemolymph ammonia was increased twofold immediately after 2 h, but returned to baseline levels after 6 and 24 h (ANOVA with Tukey’s pairwise comparisons, P < 0.05, N = 6, Fig. 2B). Verifying microarray data from 2011^15^, abundance of *CmHiat1* mRNA as determined by qPCR in the present study was indeed also significantly down-regulated in posterior gills of green crabs exposed to 7 days of high environmental *p*CO_2_ (hypercapnia (400 Pa), 29% reduction; Student’s T-test with P < 0.05, N = 5; Supp. Fig. 3).

### Functional expression of *Carcinus maenas* Hiat1 in *Xenopus laevis* oocytes

**Figure 3:**
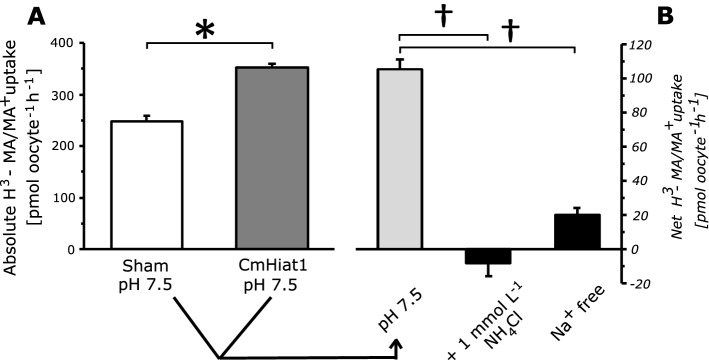
Flux studies in *Xenopus laevis* oocytes expressing *Carcinus maenas* Hiat1. (**A**) Absolute H^3^-methylamine/ammonium (MA/MA^+^) uptake of sham-injected oocytes (white bar) and oocytes expressing *Carcinus maenas* Hiat1 (dark grey bar) under control conditions (medium pH 7.5, bath [total MA/MA^+^] = 1 mmol = 10 μCi). (**B**) Net changes in MA/MA^+^ uptake (calculated as [*Carcinus maenas* Hiat1–sham]) at (i) pH 7.5 as referred to in panel (**A**), (ii) medium containing 1 mmol L^−1^ NH_4_Cl, and (iii) medium without Na^+^. Positive bars represent higher and negative bars represent lower MA/MA^+^ uptake of *Carcinus maenas* Hiat1-expressing oocytes relative to sham-injected oocytes. Asterisk indicates significant differences of MA/MA^+^ uptake between sham-injected and *Carcinus maenas* Hiat1-expressing oocytes (without sham-subtraction). Daggers indicate significant differences of MA/MA^+^ uptake between control conditions (pH 7.5 sham-subtracted, light grey bar) and treatments (black bars, sham subtracted) (Student’s T-tests with P < 0.01, N = 20). Values are presented as means ± SE. Experiments have been conducted on two different batches of oocytes (i.e., different female, different days).

Besides an Na^+^-H^+^-exchanger-3 and barely detectable levels of Rh-protein *Rhag*, *Xenopus laevis* oocytes were found to endogenously express *XlHiat1* mRNA but not *Rhbg* and *Rhcg* (Supp. Fig. 4). Consequently, ^3^H-methylamine/ammonia (MA/MA^+^) uptake potentially mediated by XlHiat1 was observed in the sham-injected oocytes at a relatively high background level of approximately 248 pmol oocyte^−1^ h^−1^ at a bath pH of 7.5. When expressing CmHiat1, MA/MA^+^ uptake increased significantly 1.4-fold under these conditions (Student’s T-test with P < 0.05, N_sham_ = 19, N_CmHiat1_ N = 18; Fig. 3A), resulting in a net (sham-subtracted) increased uptake of 104.9 ± 6.1 pmol oocyte^−1^ h^−1^ (Fig. 3B).

External NH_4_Cl added to the medium (1 mmol L^−1^) completely and competitively inhibited MA/MA^+^ uptake by CmHiat1 (Student’s T-test with P < 0.001, N = 18; Fig. 3B). In a Na^+^-free environment, MA/MA^+^ uptake by CmHiat1 decreased by 83% (Student’T-test with P < 0.001, N_sham_ = 18, N_CmHiat1_ = 20; Fig. 3B).

Oocytes pre-loaded with MA/MA^+^ and returned into regular medium without MA/MA^+^ released MA/MA^+^ back into the medium. The CmHiat1-expressing oocytes did so at significantly higher rates (38.5 ± 1.1% of preload levels/hour) than sham-injected oocytes (14.1 ± 3.8% of preload levels/hour) (Mann–Whitney Test with P < 0.005, N = 20; Supp. Fig. 5). While we obtained very similar data with both different batches of oocytes, Fig. 3 only represent the data from the second batch of oocytes. This is because in the first attempt we failed to measure the background activity of the solution necessary for the calculation from CPM to flux.

### Oocyte NH_4_^+^ and K^+^ flux measured by scanning ion-selective microelectrode technique (SIET)

**Figure 4:**
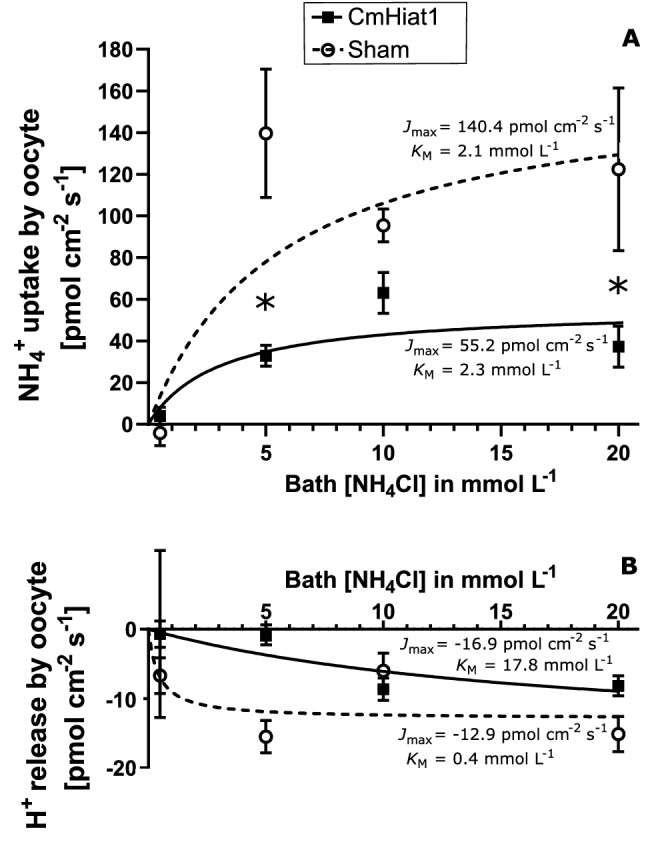
SIET measurements of net fluxes in *Xenopus laevis* oocytes expressing *Carcinus maenas* Hiat1. (**A**) NH_4_^+^ uptake (positive values, bath to oocyte) and (**B**) H^+^ release (negative values, oocyte to bath) kinetics of sham-injected (dotted line, empty circles) and *Carcinus maenas* Hiat1-expressing (solid line, black squares) oocytes in baths with increasing NH_4_Cl concentration. Asterisks in (**A**, **B**) denote significant differences between sham-injected and *Carcinus maenas* Hiat1-expressing oocytes with regards to either NH_4_^+^ or H^+^ flux at the given bath [NH_4_Cl] (two-way ANOVA with Bonferroni multiple comparisons, P < 0.05, N = 5). Values are presented as means ± SE. Experiments have been conducted on two different batch of oocytes (i.e., different female, different days).

Two-way ANOVA on SIET measurements for NH_4_^+^ uptake kinetics revealed the factors “oocyte type” (i.e., sham-injected *vs*. CmHiay1-expressing) and “bath [NH_4_Cl]” to have a significant effect on Hiat1-mediated NH_4_^+^ flux, but not their interaction. NH_4_^+^ uptake by both sham-injected and CmHiat1-expressing oocytes increased with increasing bath NH_4_Cl concentration and followed a Michalis-Menten kinetics (Fig. 4A). *K*_M_ values for both were comparable with 2.1 mmol L^−1^ for sham-injected and 2.3 mmol L^−1^ CmHiat1-expressing oocytes, while *J*_max_ values differed considerably with 140.4 pmol cm^−1^ s^−1^ for the former and 55.2 pmol cm^−2^ s^−1^ for the latter. At 5 and 20 mmol L^−1^ bath [NH_4_Cl], NH_4_^+^ uptake was significantly higher in sham-injected oocytes compared to CmHiat1-expression oocytes (Bonferroni post-hoc test with P < 0.05, N_sham_ = 5–6, N_CmHiat1_ = 5–6, Fig. 4A). While we obtained very similar data with both batches of oocytes, Fig. 4 depicts only the data from the second batch of oocytes. This is because the method used with the first batch did not allow to calculate average *K*_M_ and *J*_max_ values, but only a single value for all the oocytes.

SIET kinetics on the release of H^+^ from the oocyte into the medium of sham-injected and CmHiat1-expressing oocytes exhibited no significant difference between the two curves (two-way ANOVA with P < 0.05, N_sham_ = 5, N_CmHiat1_ = 5, Fig. 4B), although *K*_M_ values appeared considerably different with 17.8 mmol L^−1^ and 0.4 mmol L^−1^ for sham-injected and CmHiat1-expresssing oocytes, respectively. *J*_max_ values were similar with − 16.9 pmol cm^-1^ s^-1^ for sham-injected and − 12.9 pmol cm^−1^ s^−1^ for CmHiat1-expressing oocytes (Fig. 4B).

In contrast to Fig. 4A ,  B, the oocyte flux study in Supp. Fig. 2B was conducted at constant 0.1 mmol L^−1^ external NH_4_Cl, a concentration in which endogenous NH_4_^+^ uptake pathways in *Xenopus* oocytes are inactive^26^. No difference in uptake of K^+^ between sham-injected and CmHiat1-expressing oocytes was detected when measured in a modified OR2 bath containing 2.5 mmol L^−1^ KCl, while significantly higher NH_4_^+^ secretion was maintained in CmHiat1-expressing oocytes compared to sham-injected oocytes (Student’s T-test with P < 0.05, N_sham_ = 6, N_CmHiat1_ = 6; Supp. Fig. 2B).

After pre-loading/equilibration of oocytes with 20 mmol L^−1^ NH_4_Cl for 1 h, both sham-injected as well as CmHiat1-expressing oocytes displayed an internal NH_4_^+^ concentration equivalent to the outside medium of 20.34 ± 0.70 mmol L^−1^ and 20.35 ± 1.26 mmol L^−1^, respectively (Supp. Fig. 6A). NH_4_^+^ efflux as measured by SIET from these pre-loaded oocytes into an outside medium containing 0.1 mmol L^−1^ NH_4_Cl was significantly higher in CmHiat-expressing oocytes (− 84.1 ± 8.7 pmol cm^−2^ s^−1^) compared to sham-injected oocytes (− 59.9 ± 4.5 pmol cm^−2^ s^−1^; Supp. Fig. 6B, left axis). The observed ammonia transport was independent of H^+^ movements as simultaneously measured H^+^ uptake by the oocyte did not change significantly (Supp. Fig. 6B, right axis).

## Discussion

### Hiat1 as a novel ammonia transporter

In the current study, we provide first evidence that Hiat1 is an ammonia (NH_4_^+^/NH_3_) transporter in crustaceans. This was investigated and shown by two different experimental approaches in *Carcinus maenas* Hiat1-expressing *vs*. sham-injected *Xenopus laevis* oocytes: indirectly by measuring radioactively-labelled methylamine (MA/MA^+^) flux as an established proxy for NH_3_/NH_4_^+^ fluxes^7^, as well as SIET experiments directly measuring NH_4_^+^ fluxes. First of all, both experimental approaches confirmed control (sham) frog oocytes already to take up MA/MA^+^ or NH_3_/NH_4_^+^ at a relatively high background level similar to what has been measured in other studies^28^, even without expressing *Carcinus maenas* Hiat1. This might be due to the fact that interestingly, frog oocytes endogenously express XlHiat1, as well as other potential ammonia transporters, likely promoting MA/MA^+^ and/or NH_3_/NH_4_^+^ transport along concentration gradients. The latter include Rh-protein ag/AG (Rhag/RhAG) which, however, has only been shown contribute to NH_3_/NH_4_^+^ and not MA/MA^+^ flux in this system^28^. When expressing *Carcinus maenas* Hiat1, MA/MA^+^ uptake of oocytes significantly increased compared to control oocytes, while NH_3_/NH_4_^+^ uptake in the SIET measurements decreased, rather indicating NH_3_/NH_4_^+^ excretory properties of *Carcinus maenas* Hiat1 in the latter. An explanation for this phenomenon might be a difference in chemical properties of MA/MA^+^ *vs*. NH_3_/NH_4_^+^ and consequently different transport mechanisms/substrate specificity. While MA/MA^+^ solely seems to passively follow its chemical gradient with no natural source of MA/MA^+^ in the oocytes (“channel-like”), NH_3_/NH_4_^+^ flux seems to involve an active transport component. This would allow *Carcinus maenas* Hiat1-expressing oocytes in SIET experiments to counteract passive NH_3_/NH_4_^+^ influx due to high environmental levels of NH_4_Cl and rather maintain a relatively stable excretion rate against the concentration gradient as indicated by the low *J*_max_ and relatively fast flattening of the kinetics curve at bath [NH_4_Cl] above 5 mmol L^−1^. Similar challenges with both experimental methods and potential differences in substrate transport between MA/MA^+^ and NH_3_/NH_4_^+^ have also been shown before, i.e., for the ammonia transporter Rhbg^7^. Consequently, we interpreted our MA/MA^+^ data predominantly in a functional context, whereas SIET data is considered more relevant for the physiological context. Recently, an isoform of Hiat1, Hiat1b, was also verified to play a role in ammonia transport in larval zebrafish, *Danio rerio*. The respective study, however, concentrated mainly on its physiological role in this organism and did not provide details on the actual transport mechanism of the protein^29^. Additionally, the recent identification of two Hiat isoforms, LpHiat1α and LpHiat1β, in the horsehoe crab *Limulus polyphemus*, supports our findings of Hiat1 to promote ammonia excretion as demonstrated in our SIET experiments^30^.

### Hiat1 likely promotes Na^+^-dependent NH_3_/NH_4_^+^ excretion

Addition of 1 mmol L^−1^ NH_4_Cl to the incubation medium was able to completely abolish *Carcinus maenas* Hiat1-mediated MA/MA^+^ transport, verifying NH_3_/NH_4_^+^ as a substrate for Hiat1. While this does not provide further information on the direction of the flux, it clearly demonstrates the higher substrate affinity for the former over the latter. In fact, when preloaded with MA/MA^+^ *Carcinus maenas* Hiat1 promoted excretion of this substrate into the bath, indicating that MA/MA^+^ transport is solely dependent on concentration gradients in this set of experiments (see also explanation above). Similarly, when preloaded with NH_4_Cl, *Carcinus maenas* Hiat1-expressing oocytes excreted ammonia at a higher flux rate compared to control oocytes, despite exhibiting the same initial internal NH_4_^+^ concentration, again verifying *Carcinus maenas* Hiat1 to be involved in ammonia transport.

In contrast to our MA/MA^+^ experiments, however, we can conclude from our more physiological relevant SIET kinetics experiments, that *Carcinus maenas* Hiat1 promotes likely a secondary active NH_3_/NH_4_^+^ excretion from oocytes into the bath even against a concentration gradient. Furthermore, our experiments showed this *Carcinus maenas* Hiat1-mediated ammonia excretion to be (1) independent of protons but (2) dependent on extracellular Na^+^. Additionally, our data revealed that *Carcinus maenas* Hiat1 does not promote K^+^ transport, as elaborated on below.

As a major route for ammonia excretion in gills^31^ and kidney^32^ of freshwater fish as well as the mammalian kidney^33^, it has been suggested that ammonia (in this case NH_3_) in these epithelia is excreted via Rhesus glycoproteins partaking in “ammonia trapping” by titration with H^+^ to form NH_4_^+^. Our SIET measurements, however, did not detect any changes in [H^+^] in close proximity of the oocyte which would have been expected if titration of NH_3_ to NH_4_^+^ had been applied at the oocyte surface. Consequently, it is indeed likely NH_4_^+^, and not NH_3_, that was excreted by *Carcinus maenas* Hiat1.

Our findings therefore set Hiat1 apart from Rhesus glycoproteins which have been suggested to rather be gas channels transporting NH_3_ either directly (Rhcg), or indirectly by recruiting NH_4_^+^ to a specific site and then de-protonating it (RhAG and Rhbg)^34,35^. Interestingly, however, *Carcinus maenas* Hiat1 has a comparable *J*_max_ (55.2 pmol cm^−2^ s^−1^) to Rhcg in rainbow trout (63.3 pmol cm^−2^ s^−1^;^28^). To date, only one Rhesus-like protein associated with branchial acidified, intracellular vesicles been identified in *C. maenas*^17,36^, in contrast to its localization in cell membranes in vertebrates^37^. *Carcinus maenas* Hiat1 might therefore complement Rhesus-like protein function as a candidate for a membrane-associated NH_4_^+^ excretion.

While the *Carcinus maenas* Hiat1-associated NH_4_^+^ excretion is clearly dependent on external [Na^+^] and diminishes in its absence, the actual mechanism is less clear at this point. Generally, movements of Na^+^ contribute substantially to the oocytes’ cellular membrane potential of ca. − 50 mV^38^. It hence can be hypothesized that lack of external Na^+^ might lessen the membrane potential and subsequently decrease the electrochemical gradient for the excretion of cations, including NH_4_^+^. It also cannot be excluded that *Carcinus maenas* Hiat1 itself acts as an Na^+^/NH_4_^+^-antiporter as proposed for Na^+^/H^+^-exchanger-mediated Na^+^/NH_4_^+^ transport in the mammalian kidney^37^. Further experiments will be needed to ultimately clarify the role of Na^+^ in Hiat1 function.

With their similar size and charge, NH_4_^+^ and K^+^ have been shown to be transported by the same transporters in many cases, for example the Na^+^/K^+^-ATPase^39,40^ or K^+^-channels like the hyperpolarization-activated cyclic nucleotide gated Na^+/^K^+^ channel HCN2 in mammals^37,41^ and green crabs^16^. In the thick ascending limb of the mammalian kidney, apical NH_4_^+^ absorption has mainly been associated with substitution of NH_4_^+^ for K^+^ in the kidney-specific Na^+^-K^+^-2Cl^–^ cotransporter NKCC2 with minor roles for K^+^/NH_4_^+^ exchange and Ba^2+^-sensitive K^+^-channels^37^. Our SIET experiments, however, showed that *Carcinus maenas* Hiat1 does not promote K^+^ transport and might hence be substrate-specific for NH_4_^+^.

### ***Carcinus maenas*** Hiat1 has a physiological function in ammonia regulation/handling in response to environmental disturbances

Besides the obvious disruptive effects of high environmental ammonia^42^, also high environmental *p*CO_2_ (hypercapnia) has been shown to effect ammonia homeostasis in crustaceans^14,43^. In response to the latter, ammonia has been discussed to be utilized in addition to the carbonate system to offset disturbances of the hemolymph’s pH equilibrium in *C. maenas*^14^. *Carcinus maenas Hiat1* was indeed the only identified ammonia-related transcript that showed a significant decrease in mRNA abundance in response to hypercapnia (7 days, 400 Pa) in the microarray study on green crabs by Fehsenfeld et al.^15^, which we here confirmed by quantitative PCR. In this context, the down-regulation of branchial *Carcinus maenas Hiat1* mRNA abundance is likely correlated with the observed elevated hemolymph ammonia levels due to decreased excretion, potentially contributing to the hemolymph’s buffering capacity. Similarly, when DrHiat1b protein was absent due to *DrHiat1b* knock-down in zebrafish larvae, whole animal ammonia excretion significantly decreased^29^.

With regards to high environmental ammonia (HEA, 1 mmol L^−1^), green crabs experienced a two-fold increase in hemolymph ammonia within the first 2 h of acclimation. However, in this case, the up-regulation of *Carcinus maenas Hiat1* mRNA abundance then after 6 h and 24 h correlates with a drop of hemolymph ammonia back to control levels. In consequence, (*Carcinus maenas*) Hiat1 might prove important not only (directly) for ammonia homeostasis, but also (indirectly) for general acid–base homeostasis and regulation.

### Important role for Hiat1 throughout evolution

The high level of evolutionary conservation for Hiat1 demonstrates the overall importance of this transporter likely in the detoxification of nitrogen waste for animals and/or with NH_4_^+^ being a major inorganic nitrogen source promoting growth in plants. Furthermore, the fact that mRNA expression of this gene can be found naturally in frog oocytes suggests that—as a maternal gene—it is ensured to be passed on, again indicating its importance. In frogs in particular, a way for ammonia detoxification would also be especially crucial early on in development before a potential switch to urea excretion in the absence of a water environment^44^. In the latter case, Hiat1 might contribute considerably to ammonia detoxification of the oocytes in the absence of the majority of Rhesus glycoproteins and therefore have a similar role to AMT in insect sperm^12^.

## Conclusion and future directions

In summary, here we identified a novel evolutionarily conserved ammonia transporter, likely able to directly mediate a Na^+^-linked secondary active NH_4_^+^ transport against concentration gradients. Due to its conservation in the animal kingdom and its ubiquitous tissue expression, this NH_4_^+^ transporter appears to provide a fundamental pathway for cellular ammonia detoxification. This might prove especially important not only in ammonia transporting epithelia as found e.g., in nephrons and gills, but also more generally for fertility, considering the malformation of sperm cells in *Hiat1/Hiat1* mutants in mice. With ammonia being a key acid–base equivalent (i.e., NH_3_ as base and NH_4_^+^ as acid component), Hiat1 is likely also a major player in cellular and systemic pH regulation and therefore essential for general physiological processes and homeostasis.

Future experiments are needed to investigate the actual link to Na^+^ transport and determine the electrogenicity of the transporter, respectively. Furthermore, it would be desirable to elucidate likely Hiat-mediated shifts also in intracellular pH and—especially considering its dependency of Na^+^ transport, i.e., connection to actions of the Na^+^/K^+^-ATPase—its potential effect on transmembrane potential.

## Supplementary Information

Supplementary Information.

## Data Availability

The data that support the findings of this study are openly available in FigShare at 10.6084/m9.figshare.21445677. The full sequence of *CmHiat1* can be accessed at GenBank under the accession no. MT371392.1 (https://www.ncbi.nlm.nih.gov/nuccore/MT371392.1).

## Abbreviations

aa: Amino acid
AMT: AMmonia Transporter
ANOVA: ANalysis Of Variance
CHCl: CHolin Chloride
CDK 1/2/5: Cyclin-Dependent Kinases 1/2/5
CK I/II: Creatine Kinases I/II
*Cm*: Carcinus maenas
cRNA: Copy RiboNucleuc Acid
*EF1α*: Elongation Factor 1α gene as reference gene for qPCR
GSK3: Glycogen Synthase Kinase 3
HEA: High Environmental Ammonia
Hiat1: Hippocampus Abundant Transcript 1 protein (*Hiat1* in italic refers to gene)
*HsGLUT3*: *Home sapiens* GLUcose Transporter 3 gene
*J*_max_: Maximum flux rate (Michaelis–Menten-Kinetics)
kDa: Kilo Dalton
*K*_M_: Michaelis Menten constant
MA/MA^+^: ^3^H-MethylAmine/ammonia
MEP: MEthylammonium Permease proteins
MFS: Major Facilitator Superfamily of transport proteins
Mfsd14a: Renamed mammalian equivalent to Hiat1
mRNA: Messenger RiboNucleuc Acid
NH_3_/NH_4_^+^: Ammonia gas/ammonium ion (ammonia as used in the manuscript refers to the sum of both)
OR2: Oocyte Ringer solution 2
ORF: Open Reading Frame
p38MAPK: P38 Mitogen-Activated Protein Kinase
Pa: Pascal
PKA/PKC: Protein Kinase A/C
qPCR: Quantitative Polymerase Chain Reaction
*Rbs-3*: Ribosomal gene 3 (reference gene for qPCR)
RLM-RACE: RNA Ligase-Mediated Rapid Amplification of cDNA Ends
RNAi: RNA interference technique
SE: Standard Error
SIET: Scanning Ion-selective Electrode Technique
*Xl*: *Xenopus laevis*
