# Supplementary material for: Hiat1 as a new transporter involved in ammonia regulation
Source: Sci Rep. 2023 Mar 17;13:4416. doi: 10.1038/s41598-023-31503-0 (PMC10023664; doi:10.1038/s41598-023-31503-0)

**Supplemental material**

**
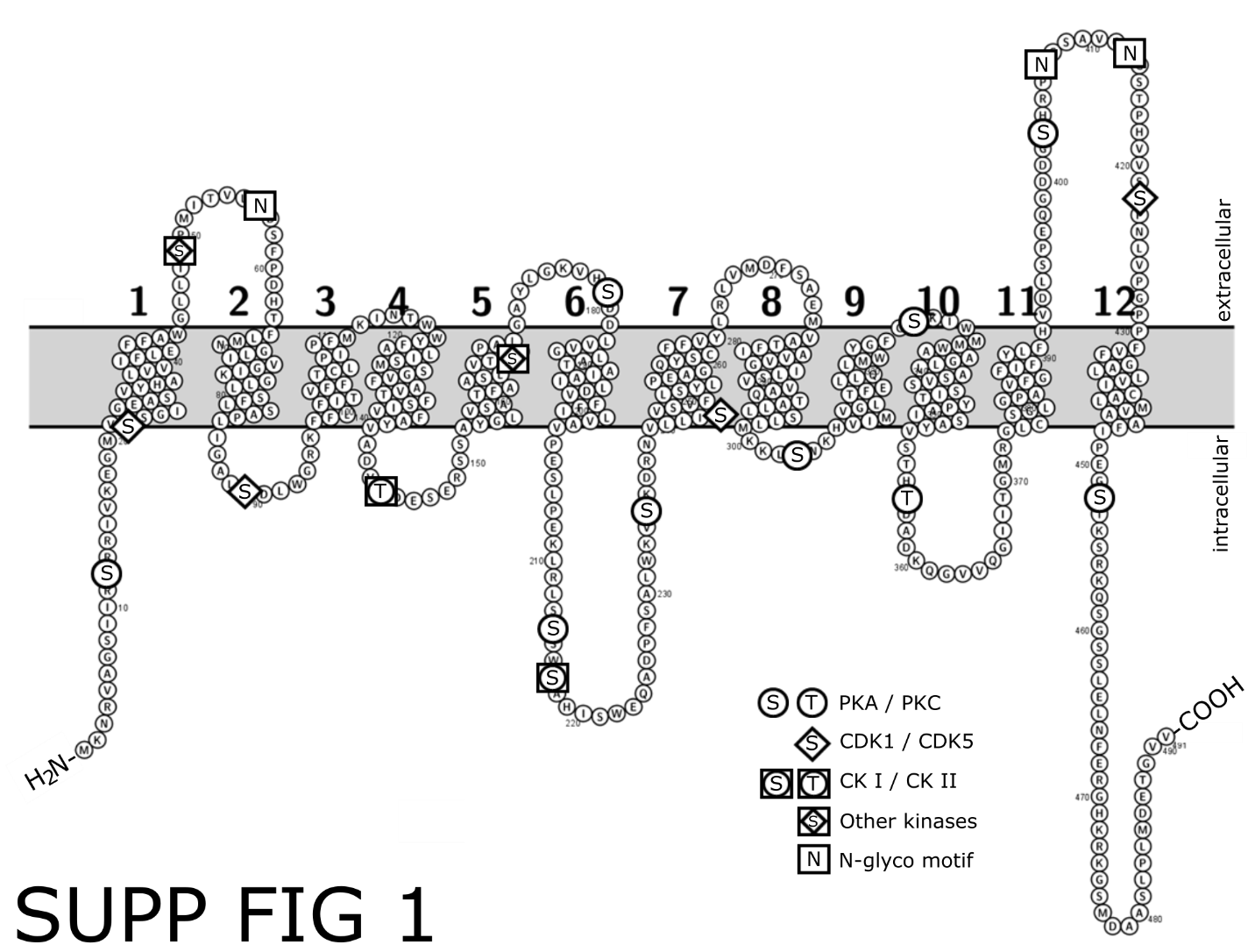
**

**Supplemental Figure 1. Structural analysis of CmHiat1.** Shown are the predicted 12 transmembrane domains as calculated by Protter (https://wlab.ethz.ch/protter). Emphasis is on potential phosphorylation sites for protein kinases A and C (PKA/PKC; circles), cyclin-dependent kinases 1 and 5 (CDK1/CDK5; diamonds), creatine kinases I and II (CKI/CKII; circles in squares), p38 mitogen-activated protein kinase and Glycogen synthase kinase 3 (other kinases; diamonds in squares), as well as the N-Glyco motif (squares).


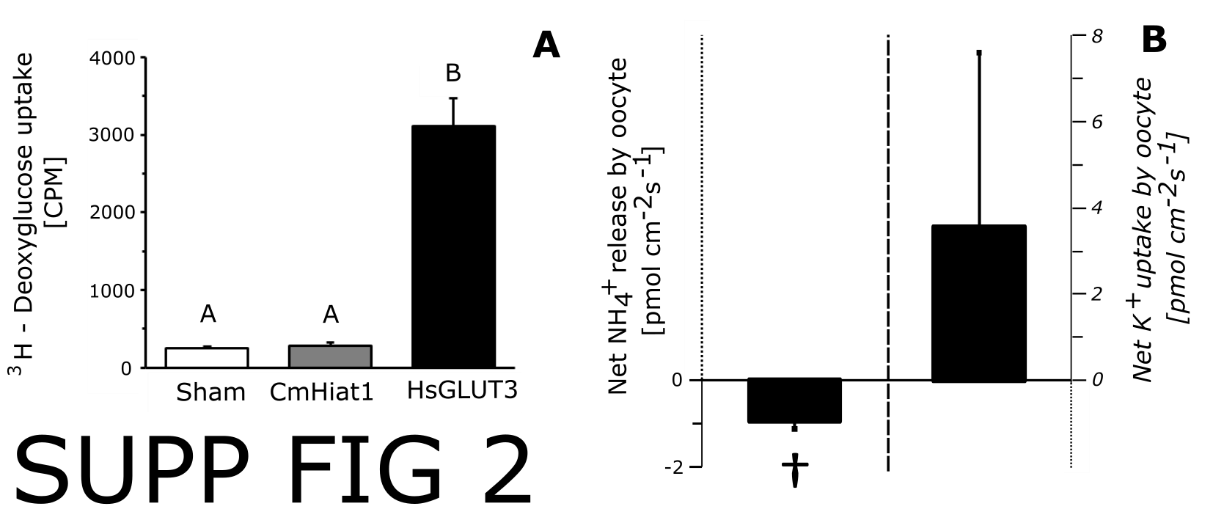


**Supplemental Figure 2. Oocyte fluxes for alternative CmHiat1 substrates.** (A) H^3^-Deoxyglucose uptake as a measure of glucose transport in sham-injected, CmHiat1-expressing and HsGLUT3-expressing *Xenopus laevis* oocytes. HsGLUT3 was included as a positive control. Letters denote significant differences between sham-injected and transgenic oocytes (Kruskal-Wallis Test with Mann-Whitney pairwise comparison and Bonferroni correction, P < 0.05, N = 18). (B) Simultaneously measured NH_4_^+^ release (left axis) / K^+^ uptake (right axis, italic) from sham-injected or CmHiat1-expressing oocytes into / from the bath (OR2 buffer containing 0.1 mmol L^-1^ NH_4_Cl) in SIET experiments. Bars in (B) represent sham subtracted ([CmHiat1 – sham]) net fluxes. OR2 buffer was modified to contain 2.5 mmol L^-1^ KCl. The dagger denotes a significant difference between sham-injected and CmHiat1-expressing oocytes before sham subtraction (Student’s t-test, P < 0.05; N = 6). All values are presented as means ± SE. Experiments have been conducted on two different batches of oocytes (*i.e*., different female, different days).


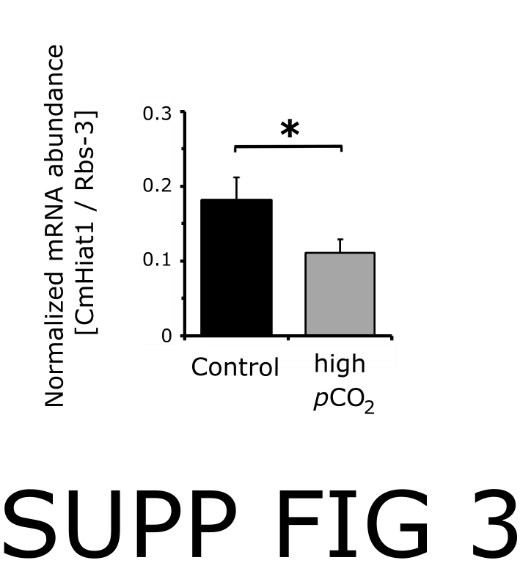


**Supplemental Figure 3. Analysis of *CmHiat1* mRNA abundance in response to hypercapnia by quantitative PCR.** mRNA abundance of *CmHiat1* in posterior gill #7 under control conditions and after 7d-acclimation to 400 Pa *p*CO_2_. Asterisk denotes significant difference in mRNA abundance as determined by Student’s t-test (P < 0.05, N = 5). Values are presented as means ± SE.


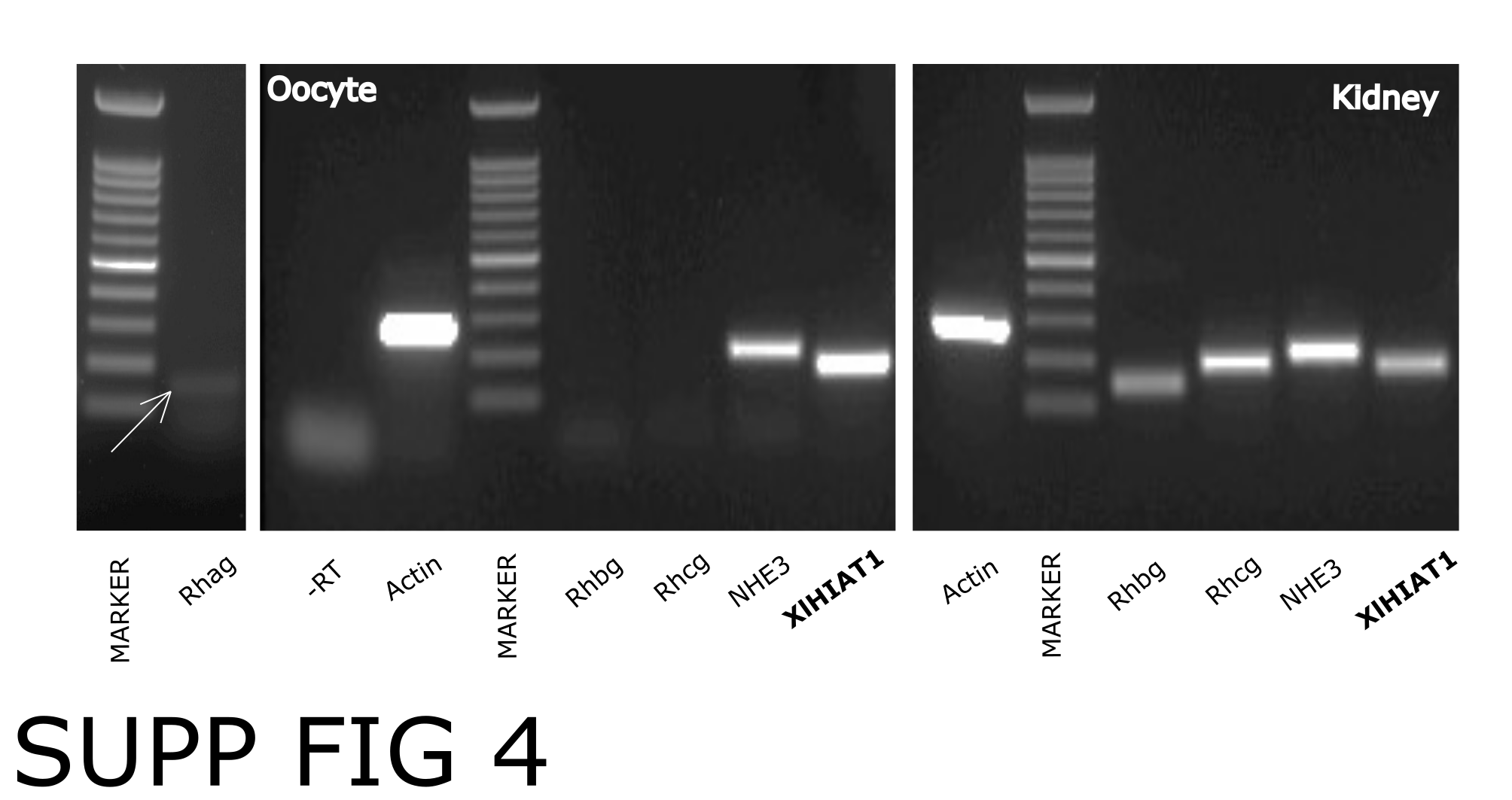


**Supplemental Figure 4. mRNA abundance of *CmHiat1* and other epithelial transporters in *Xenopus laevis* oocytes and kidney.** Ethidium bromide gel electrophoresis of Rhesus-glycoproteins *Rhbg* and *Rhcg*, Na^+^/H^+^-exchanger (*NHE3*) and *XlHIAT1* (*MFSD14*), as well as actin as a positive control. Samples were run on three separate gels as indicated by white spacing in between panels. -RT, negative control. Primers used as listed in Supp. Table 2. Marker range from 100 – 1500 bp with 500 bp indicated as a bold signal. White arrow was included to accentuate band for Rhag. For uncropped versions of the pictures see the end of this document; processing of the images as detailed in material and methods.

**
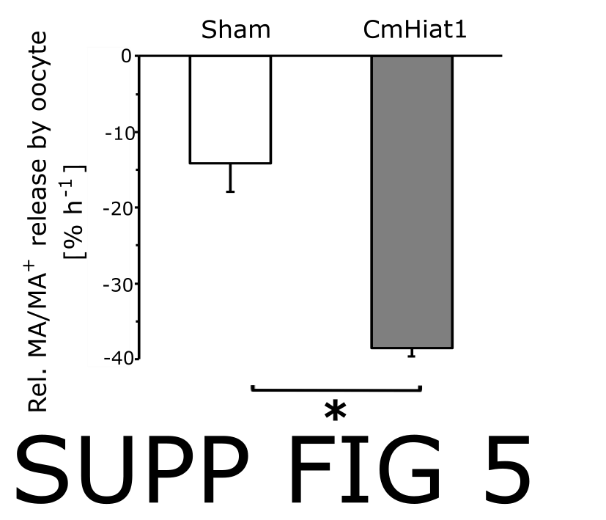
**

**Supplemental Figure 5. Release of H^3^-Methylamine/ammonium (MA/MA^+^) of pre-loaded *Xenopus laevis* oocytes.** Oocytes were incubated in medium containing MA/MA^+^ for 0 min and 60 min, washed and 20 oocytes measured for each time point. Values (means ± SE, N = 20) are expressed as percent of the 0 min value. Asterisk denotes significant difference (Mann-Whitney-Test with P < 0.001, N = 20). Experiments have been conducted on two different batch of oocytes (*i.e*., different female, consecutive days).


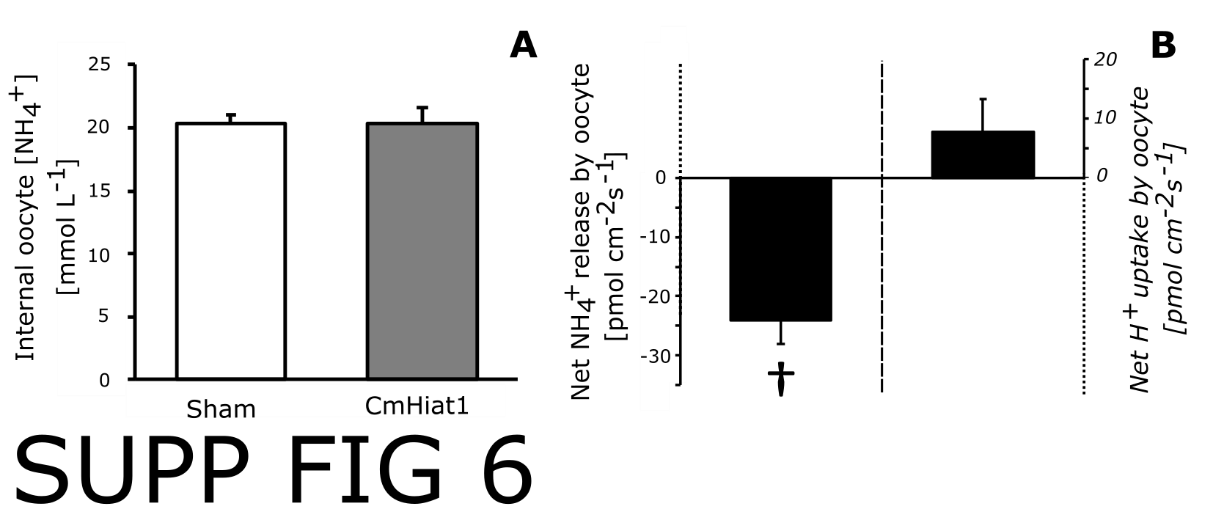


**Supplemental Figure 6. NH_4_^+^ pre-loading and subsequent release of ammonia and protons in sham-injected and CmHiat1-expressing oocytes.** Oocytes were incubated in medium containing 20 mmol L^-1^ NH_4_Cl for 60 min before either (A) their internal NH_4_^+^ content was measured by a gas-selective electrode, or (B) the simultaneous release of NH_4_^+^ (left y-axis) and uptake of H^+^ (right y-axis, italic) was measured by SIET. Dagger denotes significant difference in the sham-subtracted value (Student’s t-test with P < 0.05, N = 16 for Supp Fig. 6A and N = 5 for Supp. Fig. 6B). All values are represented as means ± SE. Experiments have been conducted on two different batches of oocytes (*i.e*., different female, consecutive days).

Supp. Table 1. Information on HIAT1 protein as included in the phylogenetic analysis.

| **Species** | **Phylum** | **Accession no.** |
| --- | --- | --- |
| *Sporisorium reilianum* | Fungi | CBQ72270.1 |
| *Coccomyxa subellipsoidea* | Plants (Algae) | XP_005647774.1 |
| *Helianthus annus* | Plants | XP_035835254.1 |
| *Rosa chinensis* | Plants | XP_024163207.1 |
| *Citrus sinensis* | Plants | XP_006485218.1 |
| *Camellia sinensis* | Plants | XP_028056471.1 |
| *Trichoplax adherens* | Placozoa | XP_002113942.1 |
| *Caenorhabditis elegans* | Nematoda | NP_493670.2 |
| *Amphimedon queenslandica* | Porifera | XP_011404646.1 |
| *Toxocara canis* | Nematoda | KHN77510.1 |
| *Strongylocentrotus purpuratus* | Echinodermata | XP_780651.4 |
| *Asterias rubens* | Echinodermata | XP_033636693.1 |
| *Nematostella vectensis* | Cnidaria | XP_032242078.1 |
| *Acropora millepora* | Cnidaria | XP_029189426.1 |
| *Hydra vulgaris* | Cnidaria | XP_002163804.2 |
| *Drosophila melanogaster* | Arthropoda | NP_647771.1* |
| *Daphnia pulex* | Arthropoda (Insecta) | EFX90342.1 |
| *Ixodes scapularis* | Arthropoda (Insecta) | XP_002433605.1 |
| *Bombus terrestris* | Arthropoda (Insecta) | XP_003401551.1 |
| *Pollicipes pollicipes* | Arthropoda (Crustacea) | XP_037092469.1 |
| *Carcinus maenas* | Arthropoda (Crustacea) | this study |
| *Penaeus monodon* | Arthropoda (Crustacea) | XP_037798249.1 |
| *Chionoecetes opilio* | Arthropoda (Crustacea) | KAG0729092.1 |
| *Ciona intestinalis* | Tunicata | XP_009858603.1 |
| *Rhincodon typus* | Elasmobranchii | XP_020385853.1 |
| *Amblyraja radiata* | Elasmobranchii | XP_032883834.1 |
| *Scyliorhinus canicula* | Elasmobranchii | XP_038650699.1 |
| *Danio rerio* | Chordata (Teleostei) | AAH97075.1 |
| *Carassius auratus* | Chordata (Teleostei) | XP_026091418.1 |
| *Oncorhynchus mykiss* | Chordata (Teleostei) | XP_021417973.1 |
| *Salmo salar* | Chordata (Teleostei) | XP_014071494.1 |
| *Xenopus laevis* | Chordata (Amphibia) | NP_001087834.1 |
| *Apteryx rowi* | Chordata (Aves) | XP_025923080.1 |
| *Spheniscus humboldti* | Chordata (Aves) | KAF1429759.1 |
| *Calypte anna* | Chordata (Aves) | KFO98688.1 |
| *Taeniopygia guttata* | Chordata (Aves) | XP_002188818.1 |
| *Orcinus orca* | Chordata (Mammalia) | XP_033289584.1 |
| *Mus musculus* | Chordata (Mammalia) | NP_032272.2 |
| *Rattus norvegicus* | Chordata (Mammalia) | NP_001099937.1 |
| *Homo sapiens* | Chordata (Mammalia) | EAW72976.1 |

##### Accession numbers are according to GenBank. * indicates a sequence from a transcriptome assembly.

Supp. Table 2. *Xenopus laevis* primers as used in this study.

| **Gene** | **Primer** | **Sequence (5’🡪 3’)** | **Product size (bp)** | **GenBank accession no. (Ref)** |
| --- | --- | --- | --- | --- |
|  |  |  |  |  |
| Actin | Forward  Reverse | TGCCTTGCCCCATGCTATCCT  GGAAGAGTGCCTCTGGGCATCTG | 281 | NM Xl 001172163 |
| NHE3 | Forward  Reverse | AAATTTGGTCGCAAGCATGT  CCCCTTGCTGTCTGCTAAAG | 219 | XM_031903807.1* |
| Rhbg | Forward  Reverse | ATTGGAGCTATTGTGGCACTCT  TCCCAAAGCTACCAGCAGTG | 144 | BC078079 (35) |
| Rhcg | Forward  Reverse | TGGGAAGATCCTCATTGGAG  CCACCAGCATCAATTACGTG | 195 | NM Xl 001095084 (35) |
| XlHiat1 | Forward  Reverse | AGCTATTGGCGCTTACCTGG  GCAAAAGGGTCTGCTTGCTC | 180 | NM_001092143.1 |

* These primers are based on the NHE3 sequence of *X. tropicalis*.

**Uncropped images of Supplemental Figure 4**

Panel 1: Panel 2 + 3:


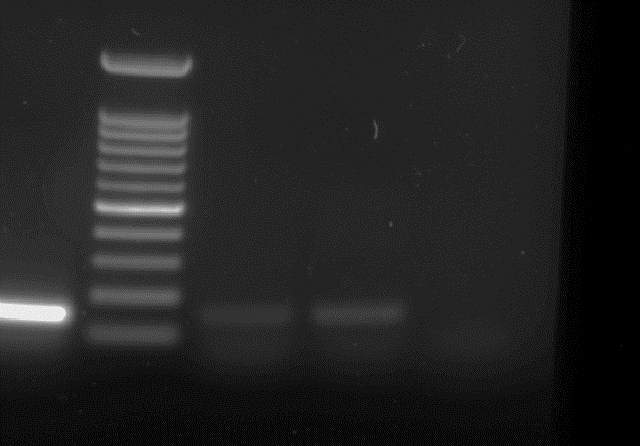


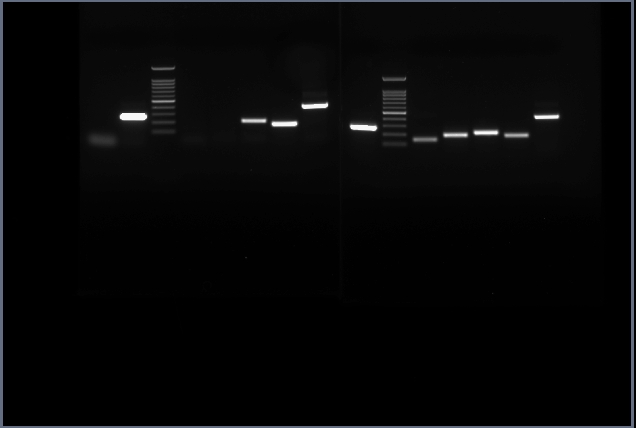

Supplement: Supplementary file 1 — Supplementary Information. [file 41598_2023_31503_MOESM1_ESM.docx]
